# Incidence and Risk Factors of Postoperative Bleeding in Patients Undergoing Total Thyroidectomy

**DOI:** 10.3389/fonc.2020.01075

**Published:** 2020-07-23

**Authors:** Ning Sun, Danhua Zhang, Shouhua Zheng, Lijun Fu, Liwen Li, Senyuan Liu, Hongting Li, Xinguang Qiu

**Affiliations:** Thyroid Surgery Department, The First Affiliated Hospital of Zhengzhou University, Zhengzhou, China

**Keywords:** thyroid cancer, postoperative bleeding, BMI, 30-day mortality, total thyroidectomy

## Abstract

**Purpose:** Our goal was to analyze postoperative bleeding in patients undergoing total thyroidectomy and to explore the possible risk factors.

**Materials and Methods:** Patients undergoing total thyroidectomy were retrospectively enrolled, and the main study outcomes were postoperative bleeding and 30-day mortality. Univariate and multivariate analyses were used to determine the independent risk factors for postoperative bleeding.

**Results:** A total of 31,706 patients were enrolled for analysis during January 2010 and December 2018 from the Affiliated First Hospital of Zhengzhou University. Benign and malignant disease was reported in 4,521 and 27,185 patients, respectively. Postoperative bleeding occurred in 48 patients with benign disease and in 263 patients with malignant disease. There was one bleeding site in 243 patients. The branch of the superior thyroid artery was the most common arterial bleeding site, occurring in 124 patients, and the anterior jugular vein was the most common venous bleeding site, occurring in 85 patients. Multivariable analysis confirmed that hypertension, diabetes, BMI, and disease pathology were independent factors affecting postoperative bleeding in patients with benign disease and that hypertension, diabetes, BMI, operation time, tumor stage, and tracheotomy were independent factors affecting postoperative bleeding in patients with malignant disease. In patients with postoperative bleeding, there were 5 deaths; in patients without postoperative bleeding, there were 42 deaths, and the difference was significant (*p* < 0.001).

**Conclusions:** Compared with malignant disease patients, benign disease patients have a similar postoperative bleeding rate. A previous history of chemotherapy or radiotherapy has no significant effect on postoperative bleeding.

## Introduction

Surgical treatment is the most important procedure used by head and neck surgeons for curing thyroid cancers, and total thyroidectomy is an effective method in selected patients (1); the complication of postoperative bleeding is uncommon but sometimes can be lethal, and it is the main concern in the clinic. A number of researchers have aimed to clarify the incidence and potential risk factors of postoperative bleeding. Zhang et al. (2) noted an incidence of 1.5% in their 2,678 patients, and the risk of postoperative bleeding was significantly increased in individuals with a BMI > 30. Weiss et al. (3) searched the Nationwide Inpatient Sample database and reported that the rate of postoperative bleeding was 1.25%, concluding that high-volume hospitals and female sex were important for decreasing postoperative bleeding. Age > 45 years, black race, partial thyroidectomy, inflammatory thyroid disease, bleeding disorders, and chronic kidney disease were found to increase the risk of postoperative bleeding. Similar results were also reported by Dehal et al. (4) using the same database. Liu et al. (5) described their 2-year experience of thyroid re-explorative surgery; the authors reported that the occurrence of postoperative bleeding was 0.85%, and the individual risk factors included male sex, hypertension, benign pathology, and previous thyroid surgery. Different surgical procedures were included in these studies (3–12), and some underlying factors, such as the type of surgical instruments and chemotherapy, were not fully analyzed. Therefore, in the current study, the main goal was to analyze postoperative bleeding in patients undergoing total thyroidectomy and to explore the possible risk factors.

## Patients and Methods

The Zhengzhou University institutional research committee approved our study (No. ZUTS20190112), and all participants signed an informed consent agreement. All methods were performed in accordance with the relevant guidelines and regulations. All procedures performed in studies involving human participants were in accordance with the ethical standards of the institutional and/or national research committee and with the 1964 Declaration of Helsinki and its later amendments or with comparable ethical standards.

The medical records of patients undergoing thyroid surgery were reviewed during January 2010 and December 2018. The inclusion criteria were as follows: the disease was primary; total thyroidectomy was performed; age was older than 18 years; and there was no bleeding disease, including hemophilia. The following data were collected: age, sex, body mass index (BMI), smoking history, alcohol use, diabetes, hypertension, vascular disease including cerebral infarction and varicosity, operation time, postoperative pathology, chemotherapy within 4 weeks before thyroid surgery for other cancer, radiotherapy within 3 months prior to thyroid surgery for other cancer, tracheotomy, disease stage based on the 8th AJCC classification, and the time between skin closure and the first sign of postoperative bleeding. In our cancer center, low-molecular-weight heparin replacement was used for every patient taking aspirin or warfarin for vascular disease including cerebral infarction and varicosity at least 7-days prior to surgery. Drinkers and smokers were defined according to previous reports (13–15). According to the official document of China (16), a BMI > 28 was defined as obese, and a BMI ranging from 24 to 27.9 was defined as overweight. The main outcome was the occurrence of postoperative bleeding, which was defined as the event that should be surgical handle, and the second study point was 30-day mortality. All the patients were followed in an outpatient clinic or by telephone.

**Figure 1:**
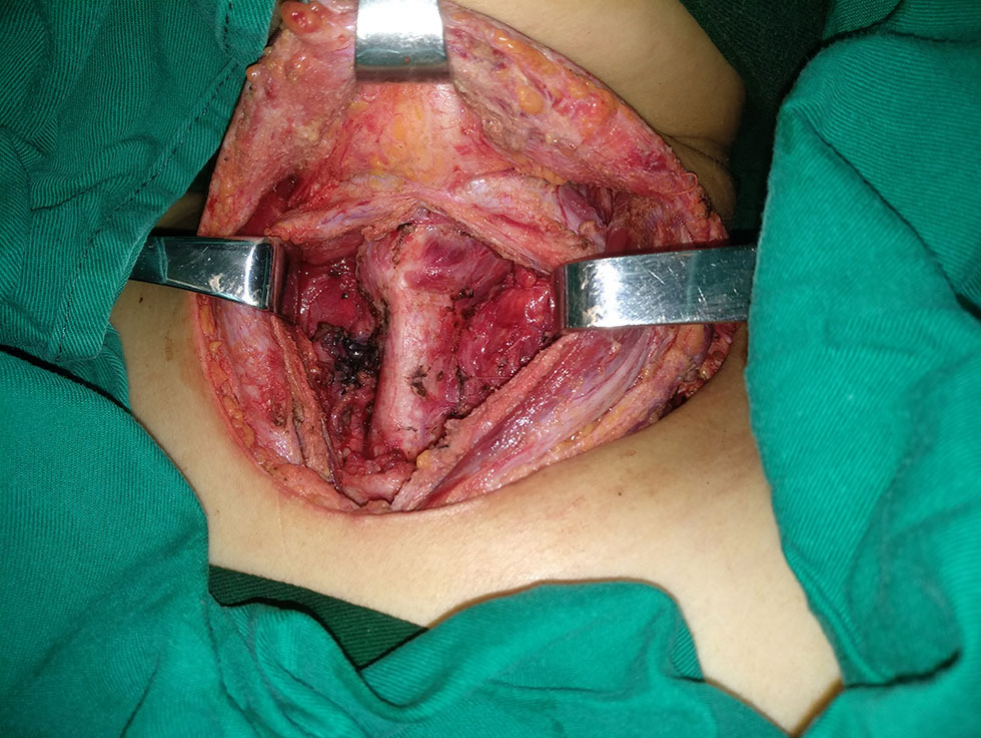
A picture showing total thyroidectomy.

Our department has 8 medical groups, every medical group has at least 6 doctors, and nearly 9,000 thyroidectomy operations are performed every year. The general principle of perioperative management was that systolic pressure was required to be not >140 mmHg for hypertensive patients before operation in most cases, the level of postoperative blood pressure was similar with the preoperative level, and fasting blood glucose was required to be not >9 mmol/L for diabetics. Antiemetic drug was selectively used postoperatively. All patients received the same surgical approach using the capsular dissection principle (2): after general anesthesia was administered, the strap muscles were divided in the middle following a collar incision. The thyroid vessels were then ligated with knot or bipolar coagulation or with an ultrasound knife. The negative pressure drainage system was used in every patient. Before wound closure, routinely increasing lung pressure was used to identify possible bleeding sites, and routine postoperative inhibition of vomiting and maintenance of proper blood pressure were used to decrease the possibility of bleeding. Hemostatics was not routinely used postoperatively (Figure 1).

The chi-square test and Student's *t*-tests were used to assess the association between potential predictive variables and the occurrence of postoperative bleeding first, and then the factors that were significant in the univariate analysis were analyzed in multivariable regression analysis to determine the independent factors. All statistical analyses were performed using SPSS 20.0, and *p* < 0.05 was considered significant.

## Results

A total of 31,706 patients undergoing total thyroidectomy were ultimately enrolled; there were 24,263 (76.5%) females and 7,443 (23.5%) males with a mean age of 50.3 (range: 19–86) years. Smokers and drinkers were noted for 4,657 (14.7%) and 4,016 (13.0%) patients, respectively. A total of 8,409 (26.5%) patients were classified as overweight, and 3,599 (11.4%) patients were classified as obese. There were 5,371 (16.9%) and 4,426 (14.0%) patients with hypertension and diabetes, respectively. A total of 1,007 (3.2%) patients had a history of previous chemotherapy, and 566 (1.8%) patients had a history of previous radiotherapy. A total of 3,506 (11.1%) patients had low-molecular-weight heparin replacement. Benign disease was reported in 4,521 (14.3%) patients, papillary thyroid carcinoma was reported in 26,961 (85.0%) patients, medullary thyroid carcinoma was reported in 130 (0.4%) patients, and thyroid follicular carcinoma was reported in 94 (0.3%) patients. 14,652 (46.2%) patients had T1/T2 disease. Lateral neck dissection was performed in 11,773 (37.1%) patients, of whom 4,134 (13.0%) underwent bilateral neck dissection. Transient and permanent hypoparathyroidism occurred in 17,895 (56.4%) and 454 (1.4%) patients, respectively. Transient and permanent hoarseness occurred in 603 (1.9%) and 224 (0.7%) patients, respectively. Surgical site infection occurred in 145 (0.5%) patients.

Postoperative bleeding occurred in 311 (1.0%) patients. In 206 (66.2%, 206/311) patients, bleeding occurred within 4 h after the operation. In 75 (24.1%, 75/311) patients, bleeding occurred between 4 and 8 h after the operation. In 30 (9.6%, 30/311) patients, bleeding occurred 8 h after the operation, and no patients required immediate tracheotomy due to postoperative bleeding. Benign disease was reported in 48 (1.1%, 48/4,521) patients, and malignant disease was reported in 263 (1.0%, 263/27,185) patients. There was a similar incidence of postoperative bleeding in patients with benign or malignant disease (*p* = 0.551). There was one bleeding site in 243 (78.1%, 243/311) patients, two bleeding sites in 30 (9.6%, 30/311) patients, and three bleeding sites in 12 (3.9%, 12/311) patients; in the remaining patients, there were no apparent bleeding sites noted.

The branch of the superior thyroid artery was the most common arterial bleeding site, occurring in 124 (62.9%) patients, followed by the branch of the inferior thyroid artery, occurring in 53 (26.9%) patients, and the branch of the transverse cervical artery, occurring in 20 (10.2%) patients. The anterior jugular vein was the most common venous bleeding site, occurring in 85 (59.9%) patients, followed by the inferior vein in 30 (21.1%) patients, the branch of the internal jugular vein in 12 (8.5%) patients, the subfascial vein in 5 (3.5%) patients, the subcutaneous tissue in 5 (3.5%) patients, and the remnant thyroid tissue of Zuckerkandl's nodule in 5 (3.5%) patients (Table 1).

**Table 1:** Distribution of bleeding sites.

**Bleeding site**	***N***
**Artery (*****n*** **=** **197)**
Superior thyroid artery	124 (62.9%)
Inferior thyroid artery	53 (26.9%)
Transverse cervical artery	20 (10.2%)
**Vein (*****n*** **=** **142)**
Anterior jugular vein	85 (59.9%)
Inferior vein	30 (21.1%)
Branch of internal jugular vein	12 (8.5%)
Subfascial vein	5 (3.5%)
Subcutaneous tissue	5 (3.5%)
Remnant thyroid tissue of Zuckerkandl's nodule	5 (3.5%)
Unknown site	26

In benign disease patients with postoperative bleeding (*n* = 48), there were 32 (66.7%) females and 16 (33.3%) males, and the mean age was 50.2 (range: 22–68) years. There were 10 (20.8%) smokers and 8 (16.7%) drinkers. Fifteen patients (31.3%) were classified as overweight, and 5 (10.4%) patients were classified as obese. Hypertension was recorded in 6 (12.5%) patients, and diabetes was recorded in 5 (10.4%) patients. Five (10.4%) patients had undergone recent chemotherapy, and 3 (6.3%) patients had undergone recent radiotherapy. Low-molecular-weight heparin replacement was used in 5 (10.4%) patients. The mean operation time was 1 (range: 0.6–2.2) h. The association between clinical pathologic variables and postoperative bleeding is presented in Table 2. Univariable analysis revealed that age, hypertension, diabetes, BMI, and disease pathology were related to the occurrence of postoperative bleeding, and multivariable analysis confirmed that hypertension, diabetes, BMI, and disease pathology were independent factors affecting postoperative bleeding (Table 2).

**Table 2:** Association between clinical pathologic variables and postoperative bleeding in patients with benign disease.

**Variables**	**Postoperative bleeding**		**Chi-square test**	**Multivariate analysis**	
	**Yes**	**No**		***p***	**HR [95% CI]**
**Age**					
<50	29 (60.4%)	2,840 (63.5%)			
≥50	19 (39.6%)	1633 (36.5%)	0.023	0.111	1.856 [0.925–3.006]
**Sex**					
Female	32 (66.7%)	2,578 (57.6%)			
Male	16 (33.3%)	1,895 (42.4%)	0.208		
**Smoker**					
Yes	10 (20.8%)	627 (14.0%)			
No	38 (79.2%)	3,846 (86.0%)	0.177		
**Drinker**					
Yes	8 (16.7%)	369 (8.2%)			
No	40 (83.3%)	4,104 (91.8%)	0.058		
**Hypertension**					
No	42 (87.5%)	4,300 (96.1%)			
Yes	6 (12.5%)	173 (3.9%)	0.011	<0.001	1.556 [1.073–2.667]
**Diabetes**					
No	43 (89.6%)	4,227 (94.5%)			
Yes	5 (10.4%)	146 (5.5%)	0.023	<0.001	1.226 [1.118–1.968]
**BMI**					
<24	28 (58.3%)	2,813 (62.9%)			
24–28	15 (31.3%)	1,212 (27.1%)		<0.001	1.666 [1.217–2.086]
≥28	5 (10.4%)	348 (7.9%)	<0.001	<0.001	2.119 [1.487–3.748]
**Chemotherapy**					
Yes	5 (10.4%)	357 (8.0%)			
No	43 (89.6%)	4,016 (92.0%)	0.591		
**Radiotherapy**					
Yes	3 (6.3%)	112 (2.5%)			
No	45 (93.7%)	4,261 (97.5%)	0.128		
LMWHR^*^					
Yes	5 (10.4%)	474 (10.6%)			
No	43 (89.6%)	3,899 (89.4%)	0.925		
**Operation time**					
≥1 h	26 (54.2%)	2,541 (5.7%)			
<1 h	22 (45.8%)	1,832 (41.0%)	0.582		
**Pathology**					
Others	20 (41.7%)	2,360 (52.8%)			
Hashimoto thyroiditis	16 (33.3%)	1,073 (24.0%)			
Follicular neoplasm	12 (25.0%)	940 (21.0%)	<0.001	<0.001	1.555 [1.064–1.994]

* *LMWHR, low-molecular-weight heparin replacement*.

In malignant disease patients with postoperative bleeding (*n* = 263), there were 215 (81.7%) females and 48 (18.3%) males, and the mean age was 50.1 (range: 19–86) years. There were 35 (13.3%) smokers and 55 (20.9%) drinkers. Sixty-two (23.6%) patients were classified as overweight, and 20 (7.6%) patients were classified as obese. Hypertension was recorded in 70 (26.6%) patients, and diabetes was recorded in 54 (20.5%) patients. Twelve (4.6%) patients had undergone recent chemotherapy, and 6 (2.3%) patients had undergone recent radiotherapy. Low-molecular-weight heparin replacement was used in 30 (11.4%) patients. The mean operation time was 2.0 (range: 1.2–6.2) h. A total of 181 (0.7%) patients required tracheotomy due to recurrent laryngeal nerve invasion by the lesion. T1–2 and T3–4 disease were noted in 97 (36.9%) and 166 (63.1%) patients, respectively. The association between the clinical pathologic variables and postoperative bleeding is presented in Table 3. Univariable analysis revealed that alcohol consumption, hypertension, diabetes, BMI, chemotherapy, operation time, tumor stage, lateral neck dissection, and tracheotomy were related to the occurrence of postoperative bleeding. Multivariate analysis confirmed that hypertension, diabetes, BMI, operation time, tumor stage, and tracheotomy were independent factors affecting postoperative bleeding (Table 3).

**Table 3:** Association between clinical pathologic variables and postoperative bleeding in patients with malignant disease.

**Variables**	**Postoperative bleeding**		**Chi-square test**	**Multivariate analysis**	
	**Yes**	**No**		***p***	**HR [95% CI]**
**Age**					
≥50	142 (54.0%)	15,222 (56.5%)			
<50	121 (46.0%)	11,700 (43.5%)	0.407		
**Sex**					
Female	215 (81.7%)	21,438 (79.6%)			
Male	48 (19.3%)	5,484 (20.4%)	0.396		
**Smoker**					
Yes	35 (13.3%)	3,985 (14.8%)			
No	228 (86.7%)	22,937 (85.2%)	0.497		
**Drinker**					
No	208 (79.1%)	23,338 (86.7%)			
Yes	55 (20.9%)	3,584 (13.3%)	<0.001	0.245	1.887 [0.896–2.006]
**Hypertension**					
No	193 (73.4%)	21,800 (81.0%)			
Yes	70 (26.6%)	5,122 (19.0%)	0.001	<0.001	1.553 [1.034–2.117]
**Diabetes**					
No	209 (79.5%)	22,701 (84.3%)			
Yes	54 (20.5%)	4,221 (15.7%)	0.031	<0.001	1.468 [1.104–2.004]
**BMI**					
<24	181 (68.8%)	16,576 (61.6%)			
24-28	62 (23.6%)	7,120 (26.4%)		<0.001	1.463 [1.078–1.998]
≥28	20 (7.6%)	3,226 (12.0%)	<0.001	<0.001	2.110 [1.547–2.997]
**Chemotherapy**					
No	251 (95.4%)	26,289 (97.6%)			
Yes	12 (4.6%)	633 (2.4%)	0.019	0.098	1.887 [0.987–2.412]
**Radiotherapy**					
Yes	6 (2.3%)	445 (1.7%)			
No	257 (97.7%)	26,477 (98.3%)	0.458		
LMWHR^*^					
Yes	30 (11.4%)	2,997 (11.1%)			
No	233 (88.6%)	23,925 (88.9%)	0.888		
**Operation time**					
<2 h	108 (41.1%)	15,812 (58.7%)			
≥2 h	155 (58.9%)	11,110 (41.3%)	<0.001	<0.001	1.557 [1.075–1.999]
**Tumor stage**					
T1+T2	97 (36.9%)	14,555 (54.1%)			
T3+T4	166 (63.1%)	12,367 (45.9%)	<0.001	<0.001	2.345 [1.567–3.669]
**Lateral neck dissection**					
No	156 (59.3%)	15,256 (56.7%)			
Unilateral	73 (27.8%)	7,566 (28.1%)			
Bilateral	34 (12.9%)	4,100 (15.2%)	<0.001	0.112	1.947 [0.913–2.664]
**Pathology**					
Papillary carcinoma	261 (99.2%)	26,700 (99.2%)			
Others	2 (0.8%)	222 (0.8%)	1.000		
**Tracheotomy**					
No	255 (97.0%)	26,749 (99.4%)			
Yes	8 (3.0%)	173 (0.6%)	<0.001	<0.001	1.765 [1.114–2.132]

* *LMWHR, low-molecular-weight heparin replacement*.

A total of 47 (0.1%) patients died within 30-days after the operation, 5 patients died of sudden serious bleeding, and 42 patients died of other systemic disease. In patients with postoperative bleeding, there were 5 (1.6%, 5/311) deaths; in patients without postoperative bleeding, there were 42 (0.1%, 42/31,395) deaths, and the difference was significant (*p* <0.001).

## Discussion

Postoperative bleeding is relatively uncommon in head and neck surgery, with an incidence of approximately 1% (17, 18); however, it is one of the most serious postoperative complications. Consistent with previous research (2–12), our study confirmed the value of hypertension, diabetes, BMI, operation time, disease pathology, and resection extent in predicting postoperative bleeding. Moreover, this was the first study to report that tracheotomy is associated with a high possibility of postoperative bleeding, and a history of previous chemotherapy or radiotherapy has no significant effect on postoperative bleeding.

The operation extent is considered to be related to postoperative bleeding. Promberger et al. (10) described an apparently higher risk of postoperative bleeding in patients undergoing bilateral near-total and total thyroidectomy compared to patients undergoing subtotal bilateral resection; a similar finding was also reported by Godballe et al. (19). In contrast, Liu et al. (20) proved that neck dissection increased the risk of postoperative bleeding. Therefore, a higher rate of postoperative bleeding in malignant disease than in benign disease should be observed due to additional resection of level 6 or lateral neck lymph nodes. However, in the current study, we noted that patients with benign disease had a similar postoperative bleeding rate to patients with malignant disease. One possible explanation for this interesting finding is that most bleeding sites are located in the thyroid gland area but not in the lateral neck area (2). Follicular neoplasms or Hashimoto thyroiditis with a rich blood supply occur in nearly half of patients with benign disease.

Arterial bleeding was more common than venous bleeding, and the most common bleeding site was the superior thyroid artery; this finding is consistent with previous reports (2, 5, 17). The management of thyroid superior vessels is an important procedure, especially in patients with Hashimoto thyroiditis or thickened blood vessels. The clamp-and-tie technique has been the most common way to divide the main vascular pedicles of the thyroid gland, but energy devices have gained increasing popularity (21). In our cancer center, multiple surgical techniques were simultaneously used during surgery, and it was difficult to analyze the potential individual role of the surgical techniques.

A limited amount of literature has reported the number of bleeding sites. It was noted that there were at least two bleeding sites in some patients, suggesting that it is necessary to continue careful re-exploration even if one bleeding site has already been found. At the same time, the fact that there might be no apparent bleeding site should be kept in mind.

Predictors of postoperative bleeding have been frequently evaluated (2–12, 17–20); common risk factors include smoking, hypertension, diabetes, high BMI, operation time, disease stage, and neck dissection, and similar findings were also observed in our results. Smoking and systemic diseases can increase vascular brittleness and decrease coagulation, and postoperative cough induced by thyroid surgery can increase blood pressure (21). A deficiency in neck extension in obese patients leads to limited exposure of the surgical field and increased operation difficulty (2). Complex thyroid surgery widens the surgical extent and increases the risk of blood vessel damage and bleeding. All of these explanations can help us better understand our outcomes.

Tracheotomy during thyroid surgery is rare due to tracheal invasion or bilateral recurrent laryngeal nerve injury. Avenia et al. (22) reported that only 43 (1.9%) of 2,165 thyroid cancer patients required tracheotomy. Similar findings were also noted in the current study. Previous studies have usually analyzed tracheotomy as an adverse consequence because of postoperative bleeding (2–12, 17–20). No authors have aimed to clarify whether tracheotomy can increase the risk of postoperative bleeding. We were the first to find that tracheotomy is associated with an additional 1.7-fold risk of postoperative bleeding. Complications occurred in 5 to 40% of tracheotomies depending on the study design, patient follow-up, and definition of the different complications; the most common complication was pneumonia (23). However, those authors typically focused on patients receiving flap reconstruction, but thyroid surgery is significantly different from pedicled or free tissue transfer operations. The following explanations might be responsible for our finding: the incised trachea is usually located within the thyroid area, which might lead to local infection; the associated cough may elevate the blood pressure, and numerous researchers have reported the close monitoring of blood pressure during the first 24 h after surgery as well as the prompt treatment of all manifestations of hypertension with appropriate drugs, which is important for decreasing the possibility of postoperative bleeding (6).

Some patients in our cancer center were transferred from other departments, and they had received chemotherapy or radiotherapy for other types of cancers before thyroid surgery. How these adjuvant treatments affect the complications of thyroid surgery remains unknown. Chemotherapy and radiotherapy are common procedures for increasing the possibility of curing cancers (15). Some authors have performed comparative studies of postoperative complications regarding the administration of preoperative chemotherapy or regarding chemotherapy regimens in patients with colorectal cancer and found that there were no clear differences (24). However, some researchers have supported that additional cardiopulmonary toxicity after esophageal cancer surgery is associated with the usage of chemoradiotherapy (25). We wish to state that a history of previous chemotherapy and radiotherapy had a limited effect on postoperative bleeding in our study. This finding could provide more information during patient conservations about operation-related complications.

Currently, 30-days after surgery is the most commonly used mortality metric and is included in major quality improvement initiatives (26–28). Sala et al. (26) described that there were no cases of 30-day mortality in their patients undergoing thyroid surgery, but the study only enrolled 61 patients. Burton et al. (27) reported that the overall 30-day mortality was 0.14% in patients undergoing thyroidectomy, but preoperative anemia was associated with a nearly 3-fold risk of death. Gupta et al. (28) noted that overall 30-day mortality was only 0.12% after thyroid and parathyroid surgery, but postoperative respiratory failure significantly increased 30-day morbidity and mortality. Similarly, we are the first to find that postoperative bleeding is related to a higher risk of 30-day mortality in our current study. A possible explanation is that patients with postoperative bleeding tend to have more systemic diseases and unhealthy habits and to be obese, and they have more extensive surgical procedures.

The limitations of our study must be acknowledged: first, there was inherit bias within the retrospective design; second, there were some unanalyzed factors, such as surgeon volume and the surgical instruments. In fact, all the operations were performed by experienced surgeons who had conducted at least 2,000 thyroid operations.

In summary, postoperative bleeding after total thyroidectomy is uncommon, benign disease patients have a similar postoperative bleeding rate to malignant disease patients, tracheotomy is associated with a high possibility of postoperative bleeding, and a history of previous chemotherapy or radiotherapy has no significant effect on postoperative bleeding.

## Data Availability Statement

All datasets generated for this study are included in the article/supplementary material.

## Ethics Statement

The Zhengzhou University Institutional Research Committee approved our study (No. ZUTS20190112). The patients/participants provided their written informed consent to participate in this study. Written informed consent was obtained from the individual(s) for the publication of any potentially identifiable images or data included in this article.

## Author Contributions

NS, DZ, SZ, LF, LL, SL, and HL: study design and manuscript writing. NS, DZ, SZ, and LF: studies selecting and data analysis. SZ, LF, LL, SL, HL, and XQ: study quality evaluating. SL, HL, and XQ: manuscript revising. All authors have read and approved the final manuscript.

## Conflict of Interest

The authors declare that the research was conducted in the absence of any commercial or financial relationships that could be construed as a potential conflict of interest. The handling editor declared a shared affiliation with the authors.

## Abbreviations

BMI: body mass index.
